# Visceral Leishmaniasis as an AIDS Defining Condition: Towards Consistency across WHO Guidelines

**DOI:** 10.1371/journal.pntd.0002916

**Published:** 2014-07-17

**Authors:** Johan van Griensven, Koert Ritmeijer, Lutgarde Lynen, Ermias Diro

**Affiliations:** 1 Department of Clinical Sciences, Institute of Tropical Medicine, Antwerp, Belgium; 2 Public Health Department, Médecins Sans Frontières, Amsterdam, The Netherlands; 3 Department of Internal Medicine, University of Gondar, Gondar, Ethiopia; New York University, United States of America

Given the detrimental interaction between both pathogens, visceral leishmaniasis (VL)–HIV co-infection has been identified as one of the emerging challenges for VL control [1]. The epidemiological impact of HIV on VL was most strikingly illustrated by the effect of the HIV epidemic in VL-endemic countries in southern Europe, with HIV contributing to the re-emergence of VL. By early 2000, almost 2,000 cases of VL–HIV co-infection (predominantly in intravenous drug users) had been identified, with up to 50%–60% of all VL cases being HIV co-infected [2]. Fortunately, with the wide-scale introduction of highly active antiretroviral therapy (ART), a gradual decline in VL incidence has been observed in Europe over the last decade [1], [3]. Currently, the burden of VL–HIV co-infection is most apparent in some regions in East Africa, like Northwest Ethiopia, where between 20%–40% of VL cases are co-infected with HIV [1]. The problem also seems to be emerging in India and Brazil [1].

There is abundant evidence that HIV strongly affects VL treatment response, including in the East African setting. Initial parasitological failure rates are typically below 2%–3% in immunocompetent individuals, but can be as high as 50% in co-infected patients [4]. Case fatality rates in East Africa are 3–9-fold higher in HIV co-infected patients, reaching up to 15%–33% [1], [5]. Whereas relapses are uncommon (<5%) in immunocompetent individuals, this can reach 50%–60% by one year in some high-risk HIV co-infected individuals [1], [6]. ART is apparently only partially protective against relapse [6]. Importantly, repeated relapses tend to become increasingly unresponsive to treatment, and secondary prophylaxis is often required until sufficient CD4 cell count recovery has taken place (at least in areas with zoonotic transmission) [1].

At an early stage, several meetings on VL–HIV co-infection were organized by the World Health Organization (WHO) trypanosomiasis and leishmaniasis unit (Division of Tropical Diseases), and an international surveillance system was put in place. Based on the evidence available, VL was proposed—in 1995—as an Acquired Immunodeficiency Syndrome (AIDS)–defining condition requiring ART initiation irrespective of CD4 counts [7], and this has remained so since then (see Table 1) [8]. VL is now included as an AIDS-defining condition in virtually all VL-treatment guidelines for use in countries where VL–HIV is prevalent.

**Table 1:** Overview of integration of visceral leishmaniasis as an AIDS-defining condition in WHO guidelines.

Year	HIV/AIDS Program	Control of Tropical Diseases/NTD
1990	WHO clinical staging of HIV: No mention of VL	
1995		Consultative meeting on *Leishmania*-HIV co-infection: “*Leishmania*: AIDS-defining disease: any patients with co-existing HIV-infection and VL”
2003	WHO clinical staging in 2003 ART guideline: No mention of VL	
2005	Interim guideline WHO clinical staging (Africa): visceral leishmaniasis (stage 4)	
2006	WHO clinical staging in 2006 ART guideline: Atypical disseminated leishmaniasis (stage 4)	
2007	Revised WHO clinical staging: Atypical disseminated leishmaniasis (stage 4)	5th Consultative Meeting on *Leishmania*-HIV Co-infection: “VL is an AIDS-defining condition,… to start ART, irrespective of CD4 count”
2010	WHO clinical staging in 2010 ART guideline: Atypical disseminated leishmaniasis (stage 4)	WHO guideline on Leishmaniasis (2010): VL: AIDS-defining condition; …starting antiretroviral treatment, irrespective of CD4 count
2013	WHO clinical staging in 2013 ART guideline: Atypical disseminated leishmaniasis (stage 4)	

AIDS: Acquired Immunodeficiency Syndrome; ART: antiretroviral treatment; NTD: neglected tropical diseases; VL: visceral leishmaniasis; WHO: World Health Organization.

Early on in the HIV epidemic—in 1990—WHO developed an HIV clinical staging system adopted for use in low- and middle-income countries [9]. This WHO clinical staging system is widely used by the HIV medical community and forms the backbone of several important recommendations like cotrimoxazole prophylaxis and eligibility for ART. It has undergone several adaptations over the years, with the latest revision of the WHO staging system reported in 2007 [10]. This clinical staging system has also systematically been endorsed across all revised WHO ART guidelines over the last 10 years, up to the most recent version in 2013 [11]–[14].

Surprisingly, and in contrast with the latest recommendations coming out of the WHO Department of Neglected Tropical Disease and the WHO Expert Committee on the Control of Leishmaniases, VL is not systematically included as an AIDS-defining condition. Although VL appeared in the interim revised clinical staging recommendations (as a WHO stage 4 condition), the finalized version (2007) only stated “atypical disseminated leishmaniasis” as a stage 4 condition, and this was included in the 2006, 2010, and 2013 ART guidelines. This term (atypical disseminated leishmaniasis) is not clearly defined, with no presumptive clinical diagnosis proposed and as definitive diagnosis: “… histology (amastigotes visualized) or culture from any appropriate clinical specimen.” Disseminated cutaneous leishmaniasis is an established clinical entity, but “atypical disseminated leishmaniasis” is not. Strictly speaking, it is not even clear whether both VL and cutaneous leishmaniasis would be considered.

Similarly to tuberculosis–HIV co-infection, successful management and control of VL–HIV co-infection will hinge on the effective coordination of both VL and HIV programs [15]. Coordination at the global level is likely to foster successful integration of national programs [15]. For instance, in Ethiopia—which has the highest VL–HIV co-infection rates—the national VL guidelines recommend routine ART for all VL cases, but the HIV/ART guidelines will follow the international HIV clinical staging system (only mentioning “atypical disseminated leishmaniasis;” http://www.who.int/hiv/pub/guidelines/ethiopia_art.pdf). The same is true in other VL-endemic countries such as India and Uganda. In contrast, VL is an AIDS-defining condition in the national ART guidelines in Brazil and Kenya. As to Sudan and South Sudan, both “atypical disseminated leishmaniasis” and VL are included. We call upon WHO to address this inconsistency and hope this can be rectified by the next revision of the WHO guidelines.
